# The Relation of the Teeth to the Diseases of the General System

**Published:** 1889-09

**Authors:** Frank. P. Watson


### COMMUNICATIONS.

### The Relation of the Teeth to the Diseases of the General System.

BY FRANK. P. WATSON.

From the fact that truth lies round about us, all too closely to be sought—so open to our vision that 'tis hidden to our thought, and that we needs must struggle for the sight of what we always see, familiar with the strangest things, and with familiar, strange, will no doubt explain away to a certain extent, why with all the students of anatomy and physiology the teeth have remained in obscurity so long, so far as regards their importance as organs in relation to the diseases of the general system. Their position in the jaws and their relation to the surrounding stuctures it seems to me are such as to promptly arrest the attention of the most superficial student even, and confront him with their undeniable importance. The peculiarity and density of their structure is sufficiently suggestive to rank them as organs of more than ordinary importance. The same importance they maintain in relation to health, is equally sustained in their relation to disease, and the importance of their proper care and treatment can not be overestimated; this importance can not be made to be understood more forcibly perhaps than by recording a list of the evils resulting from neglecting to bestow proper care and treatment upon them.

Since my paper is upon the relation of the teeth to the diseases of the general system I will not touch upon them as regards their relation to the health of the general system.

I will first cite a few of the many local diseases and their more serious consequences as results of their neglect. Caries, periostitis, alveolar abscess, etc., these with their consequent conditions represent varied forms of disease, general as well as local, with the ever accompanying suffering and sometimes fatal results.

Caries undoubtedly produces many of the diseased conditions of the mucous membranes of the mouth, throat, and stomach with their resultant tonsilitis, laryngitis, and dyspepsia with its train of diseases, also periostitis, alveolar abscess and their evil train.

To conclusively prove that they do hold a most important relation to the diseases of the general system I will first cite instances where they acted directly, to be followed by instances where they produce diseases indirectly or reflexly; also where they in turn are the organs "yet healthy" imposed upon by diseased organs and conditions in other parts of human economy.

My first citation relative to the diseased condition of the teeth acting directly, will be that of a boy four years of age, admitted to Guy's Hospital September 30, 1874. The family history was good with the exception of a tumor in the grandmother. The boy was healthy until six weeks before his admittance, when he returned from school with a bad attack of diarrhœa, followed by a severe fright a few days later. A few days after this his left eye was noticed to be somewhat swollen which was followed two weeks later by his right eye becoming swollen also. He gradually grew worse and three weeks prior to his admission was in a drowsy state.

On admission he was delicate and vacant-looking; left eye much more prominent than the right, with thickening along the upper margin of the orbit. Beneath the edge, under the eyelid, was a cartilaginous, freely moveable body, which apparently reached backward into the orbital cavity above the eyeball while it extended downward into the eyelid. The movements of the eyeballs were perfect and sight unaffected; temperature 104.5 in the morning, 102.7 in the evening; pulse 160. Examination could give no satisfactory cause for the elevation of temperature yet though as the root of each orbit was affected, and the boy tottered as he walked, and was peculiarly torpid, the disease whatever it was, had extended from the orbital foramen to the cerebral hemisphere. The ophthalmoscope revealed only large tortuous veins with a slight hemorrhage on the outer margin of the right optic disc. The temperature remained high ; he rapidly grew worse losing control over his evacuations and died nine days after his admission.

The autopsy showed on the right side that pus extended all over the outer side forward, and appeared externally over the superciliary ridge, while it passed backward through the optic foramen and sphenoidal fissure, underneath the cavernous sinus, across the sella turcica and grooves of, or the optic commissure, through the right optic foramen and right sphenoidal fissure; all this beneath the dura mater of the base of the skull. The bone was rough and carious, and part of the body of the sphenoid infiltrated with a grumous chocolate-colored pus. Thus, it appears the right orbit had become affected after the left; pus had coursed into the spheno-maxillary fossa, and thence to the condyle of the lower jaw.

The articulation was free, but the whole of the condyle and much of the ramus was bared of its periosteum,and pus lined the inferior dental canal as far as the first molar, which was decayed lying loose in its socket and with carious bone about it.

Although the cavernous sinus of both sides were closely surrounded by pus they were unobstructed, the same of the longitudinal sinus. The frontal sinuses were normal. The lumps felt during life, over the left superciliary ridge, consisted of a tough, opaque, yellow mass, the result of an inflammatory action. Its situation was from the lachrymal duct externally to the inner margin of the orbital ring, and lay half protruding from and half within the orbit and adherent to the bone, which in its removal was bare of periosteum. There can be no doubt, from the post mortem appearance, that the source of all this destruction was a decayed tooth. It had led to alveolar abscess, thence to caries of the bone, thence to suppuration in the inferior dental canal, and thence the pus had followed the course described. It is a most striking illustration of the evils attending neglect of these important organs and the terrible results such evils may produce in an unhealthy subject. There was no history of any toothache, no swelling or any trouble about the jaws. The first was the attack of diarrhœa, followed by the swelling of first one eye then the other, thence passing into a condition of stupor, followed in a short time by death.

Then diseases of the general system that are produced reflexly by carious teeth are manifold, a record of but a few of the many reported cases will no doubt be sufficient to convey an adequate idea as to the extensive and important part the teeth play in this relation.

The illustrations I cite, I have placed for convenience in groups. The first are the varied diseases produced by carious teeth. Second, those produced by impacted molars. Third, those produced by hyper-cementosis. Fourth, those produced by pulp nodules. Fifth, those produced by dead pulps. Sixth, those produced by dentition. Seventh, those produced by the overcrowding of the teeth.

The cases I will cite under the head of carious teeth are as follows:

*Epilepsy from Carious Teeth.*—A farm-laborer was admitted to one of the hospitals of London for epilepsy. The usual remedies were tried for six weeks without effect. His mouth being examined the molar teeth of the lower jaw were found to be greatly decayed, only the roots of some remaining. During the eighteen months that followed the removal of the diseased teeth, he had not suffered from a single fit, though for many weeks previous to their removal he had had two and three a day.

A boy thirteen years old had had frequent attacks of epilepsy for eighteen months. An examination of his mouth exhibited a molar considerably decayed with a swollen gum around it, and partly growing into the cavity. Was not tender to the touch, nor did an examination cause toothache. On questioning the boy it was found the sensation he experienced before the fit was not one of pain but that of an indefinite uneasiness. He always had a fit the night this sensation was felt, which always occurred about seven or eight o'clock in the evening. The tooth was extracted and he had no recurrence of the fits.

*Epileptoid Convulsions of the Right Arm from Carious Teeth.*—An unmarried woman had had epileptoid convulsions affecting some of the muscles of the neck and right arm, of daily occurrence for four years, during which time she was under the treatment of a physician but with no appreciable benefit. There was a slight disposition to anæmia, beyond this there was no symptoms upon which to base a treatment. Appetite, digestion, circulation and general health all good, and generative organs acting normally. In inquiring into the history it was learned the first spell came on during an attack of toothache. An examination of the mouth revealed a half dozen carious teeth. These were extracted, followed by an entire absence of the convulsions.

*Chorea from Carious Teeth.*—The muscles of the face, arms and shoulder of a young lady were in constant motion for six weeks, and was increasing in severity day by day, until a carious, inferior third molar was extracted, which was followed by a cessation of the spasmodic twitching and the lady was shortly cured.

*Insanity from Carious Teeth.*—A boy aged nineteen was admitted to the New Hampshire Insane Asylum in a state of mania. It was ascertained that he had previously had a tooth extracted, that one of the roots had broken off and remained in the jaw, suppuration then took place, the pus discharged outwardly, and the boy was suddenly insane. The root was removed, the fistulous opening closed, and the patient quickly recovered from his mania.

*Trismus from Carious Teeth.*—A young lady aged seventeen showed marked symptoms of this disease. Muscular spasms, opisthotonos, and increased reflex excitability. As there was an entire absence of the usual causes of the disease, and as the patient complained of severe toothache, the thought suggested itself that the cause might lie in the diseased tooth. The tooth was extracted and the symptoms disappeared.

*General Paralysis from Carious Teeth.*—A young lady was brought to the office of a dentist. On being removed from her carriage she was supported by two servants, her entire muscular system seemed paralyzed. Her feet trailed upon the ground, her arms fell powerless when not supported, even the muscles of her tongue were paralyzed, and all efforts to speak unavailing. On examining her mouth, a third molar inferior, very carious and deeply imbedded, was found just below the coronoid process. On extracting this tooth she instantly regained her powers of speech, and communicated the fact, that ever since the extracted tooth had been forcing its way through the gum, she could date the gradual loss of power over her limbs. A month later she was seen writing a letter and possessed complete control over her limbs.

*Neuralgia of Face, Neck and Arm, with Partial Paralysis of Latter from Carious Teeth.*—A lady applied to a physician for relief from a constant aching pain on the left side of the face, neck and left arm. The pain sometimes became intensely severe. The arm had nearly lost all muscular power, the patient could not raise it to her head or squeeze any object in her hand. This condition had existed for two years, and the patient had been under medical treatment all the time. Upon examining her mouth it was found that the left inferior third molar was carious. The tooth was extracted; she immediately felt great relief, and in a few hours all the symptoms had entirely disappeared.

*Aphonia from Carious Teeth.*—The case of a patient who after an attack of toothache suddenly lost his voice. This was followed by anorexia, cough, wasting and feverishness, which lead to the belief that he was suffering from laryngeal phthisis. But the lungs were sound and there was no tenderness over the larynx. There was slight inflammation of the pharynx. All the molars of left side of inferior maxilla were decayed, and the gums and periosteum around them swollen. The teeth were removed, gums cauterized and gargles employed. On the very day the teeth were extracted the suffocation spasms abated, and on the following day the other symptoms quickly disappeared.

The next in order are the diseases produced by impacted molars. The first case that of

*Otalgia from Impacted Roots.*—A young lady had been deaf for sixteen years. Four days before the case was reported she was attacked with sharp lancinating pains in the right ear. These were of such character and constancy as to produce complete insomnia. An examination of the mouth revealed an impaction of the roots of second molar of the right side of lower jaw. These were with considerable difficulty removed, and the otalgia quickly disappeared.

*Paraplegia from Impacted Third Molars.*—A young lady had enjoyed perfect health until two years before the examination, when a weakness of the lower extremities showed itself, accompanied with an occasional impairment of vision. This condition gradually grew worse until she was unable to walk without assistance. There was complete loss of vision when she assumed the erect from the sitting posture, though in the latter she could see distinctly large objects. No apparent improvement could be observed in her condition, although all forms of medication had been tried. An examination of the mouth exhibited the lower third molars but partially erupted and pressing against the second molar sufficiently to produce a slight lateral displacement outward. These third molars were extracted. Within a week a slight improvement was observed which gradually increased until three months had passed, when every constitutional disturbance but one had entirely disappeared. The power of vision in the right eye was lost.

The next in order are those produced by hyper-cementosis.

*Insanity from Hyper-cementosis.*—The patient, a gentleman, had suffered for many years from what had been supposed to be neuralgia, which finally produced insanity. In this condition he was brought to a dentist to have a tooth extracted. With great difficulty the tooth was removed. It was sound, but growing from its roots, near the crown, was a solid tumor of cementum about the size of a filbert. The neuralgia immediately ceased and the patient was soon restored to sanity.

*Facial Neuralgia from Hyper-cementosis.*—A lady had suffered for ten years with intense facial neuralgia. Medical help failed to relieve her, and no relief was gained on severing the facial nerve on the affected side nor on the opposite one. She consulted a dentist who extracted a tooth with hypertrophied roots, and relief immediately followed the removal.

The next in order are those produced by pulp nodules.

*Facial Neuralgia.*—A lady of general good health for several weeks had been suffering with severe neuralgia in upper maxilla, right side, which radiated at times all over the face, which she attributed to several decayed teeth. Their removal, however, did not bring any favorable change. Nearly all her teeth had been extracted, and she desired to have a right central incisor, quite isolated, removed to enable her to better wear an artificial denture. This tooth was perfectly sound, yet its extraction brought instant relief. A microscopical examination of the tooth showed the presence of six nodules of secondary dentine projecting from the wall of the root portion of the pulp chamber.

A physician was obliged to give up a very extensive practice because of tic douloureaux. An extraction of one ofhis teeth, apparently sound, gave instant relief. The pulp chamber was found to contain several pulp nodules growing from the pulp wall.

*A Case of Insanity.*—A lady, aged forty, of good health and family history, began to exhibit extensive mental aberration together with periods of great melancholy. This condition lasted continuously for a year, during which time she complained from time to time of severe pains in right side of face and head. A localized tenderness of inferior right molar led a dentist to drill into the pulp cavity. The pulp was devitalized, and contained several pulp nodules. On their removal, and cleansing of the pulp chamber, all features of the insanity disappeared.

The next in order are those produced by dead pulps.

*Otalgia.*—A lady had been under the treatment of an excellent aurist for inflammation and pain in the ear, but without being benefitted. At the same time she was suffering from a pain in a right lower molar which had been filled some fifteen months before. The pulp chamber was opened into and the pulp found dead. At the moment it was penetrated the pain in the ear ceased and never returned.

The next in order are those produced by dentition.

*Nausea, Vomiting, Convulsions and Death.*—The case of a young girl of strong constitution and previous good health. The four molar teeth which complete the second dentition, developed themselves simultaneously, giving rise to intense irritation and inflammation at the angle of each jaw. Nausea and irratibility of the stomach soon set in followed by obstinate bilious vomiting. The gums which covered the teeth were lanced, but with no benefit. For several days there was general agitation, strabismus, swelling of the right eye, dilation of the pupils, followed by convulsions and death.

The next are those produced by overcrowding of the teeth.

*Trismus.*—A gentleman, aged thirty, had suffered from lockjaw and pain under right ear for twelve months. He could separate the jaws in front about a half inch. He had been under medical treatment but with no benefit. An examination of the teeth showed them to be very much crowded. On the extraction of one of the anterior molars of upper jaw the patient recovered and was never troubled again.

I will now illustrate, on the other hand, the relation of the teeth to other organs of the body by citing a few cases where the teeth, in a perfectly healthy condition and structure, have suffered because of pathological conditions in other organs. The case of a boy into whose orbit a slate pencil, one and three quarters of an inch in length, had been driven by a fall. The removal was performed without the influence of any anæsthetic, and the only complaint the little patient made during the operation was that the surgeon was making his tooth ache, which was one of the molars of the upper jaw.

A case of a man, aged thirty, who had never been troubled with neuralgia suffered for a week with severe pains in his face, though more particularly in his teeth. The pain was worse at night, effectually precluding sleep. There was no condition of the teeth that would account for the severe pain. An inquiry revealed the fact that the patient's bowels had not moved for over a week. A brisk cathartic gave prompt and complete relief.

The morbid conditions of the uterus, in both pregnant and non-pregnant state, are very common causes of odontalgia.

The case of a woman, aged thirty-five, had complained of a severe pain in a lower bicuspid for nine weeks. The pain became unbearable, and the tooth being carious was extracted, but without any relief. But by a subsequent removal and cure of an ulcerated fundus of the uterus caused an entire absence of the odontalgia.

The foregoing, which are but a few of the many reported cases, is sufficiently great in numbers and range to most conclusively convince all but the skeptic mind that there is an ever existing and extremely marked relation of the teeth with the diseases of the general system.

